# Supplementary material for: Polyketide synthase gene clusters in the ericoid mycorrhizal fungus Oidiodendron maius: genome-wide prediction, expression profiling, and functional characterization of knockout mutants
Source: Front Plant Sci. 2026 Sep 16;17:1924430. doi: 10.3389/fpls.2026.1924430 (PMC13623903; doi:10.3389/fpls.2026.1924430)
Supplement: Supplementary file 1 [file SupplementaryFile1.zip › Supplementary Materials/Supplementary Folder 1.zip!/Oidma1_AssemblyScaffolds_Repeatmasked/knownclusterblast/cluster8/jgi.p_Oidma1_37940_mibig_hits.html]

| MIBiG Protein | Description | MIBiG Cluster | MiBiG Product | % ID | % Coverage | BLAST Score | E-value |
| --- | --- | --- | --- | --- | --- | --- | --- |
| AAS90060.1 | OmtB | BGC0000009 | Aflatoxin\_biosynthetic\_gene\_cluster | 33 | 75.117370892 | 163 | 5.6e-40 |
| EAL85127.1 | O-methyltransferase,\_putative | BGC0001067 | Fumagillin\_biosynthetic\_gene\_cluster | 35 | 61.9718309859 | 134 | 3.6e-31 |
| ACZ66252.1 | APS6 | BGC0000304 | Apicidin\_biosynthetic\_gene\_cluster | 27 | 86.3849765258 | 124 | 3.7e-28 |
| ABC79591.2 | cercosporin\_toxin\_biosynthesis\_protein | BGC0001247\_c2 | Cercosporin\_biosynthetic\_gene\_cluster | 32 | 38.0281690141 | 92 | 9.2e-19 |
| AKD43502.1 | O-Methyltransferase | BGC0001409 | Dutomycin\_biosynthetic\_gene\_cluster | 33 | 36.8544600939 | 89 | 7.8e-18 |
| ADM79461.1 | O-methyltransferase | BGC0001266 | Grayanic\_acid\_biosynthetic\_gene\_cluster | 35 | 35.6807511737 | 86 | 6.6e-17 |
| ACN64847.1 | PokMT3 | BGC0001061 | Polyketomycin\_biosynthetic\_gene\_cluster | 34 | 36.1502347418 | 85 | 1.9e-16 |
| CCH26291.1 | putative\_O-methyltransferase | BGC0001158\_c2 | Bikaverin\_biosynthetic\_gene\_cluster | 33 | 36.1502347418 | 82 | 9.5e-16 |
| CCT67993.1 | bikaverin\_cluster-O-methyltransferase | BGC0000030 | Bikaverin\_biosynthetic\_gene\_cluster | 33 | 35.9154929577 | 82 | 1.2e-15 |
| AAM70356.1 | CalO6 | BGC0000033 | Calicheamicin\_biosynthetic\_gene\_cluster | 25 | 79.1079812207 | 76 | 6.8e-14 |
| sln4 | methyltransferase | BGC0001230 | Salinamides\_biosynthetic\_gene\_cluster | 30 | 38.4976525822 | 71 | 2.9e-12 |
| AFD30960.1 | CrmM | BGC0000966 | Caerulomycin\_A\_biosynthetic\_gene\_cluster | 28 | 40.6103286385 | 71 | 3.8e-12 |
| OAQ83753.1 | sterigmatocystin\_8-O-methyltransferase | BGC0001358 | Leucinostatins\_biosynthetic\_gene\_cluster | 37 | 24.6478873239 | 68 | 1.9e-11 |
| ACB47081.1 | DynO6 | BGC0001060 | Dynemicin\_biosynthetic\_gene\_cluster | 29 | 42.7230046948 | 67 | 5.4e-11 |
| PSMT3 |  | BGC0001325 | Noscapine\_biosynthetic\_gene\_cluster. | 32 | 37.79342723 | 67 | 5.4e-11 |
| AAA83421.1 | RdmB | BGC0000265 | Rhodomycin\_biosynthetic\_gene\_cluster | 31 | 39.6713615023 | 60 | 5.1e-09 |
| KDN80042.1 | methyltransferase | BGC0001074 | Cosmomycin\_D\_biosynthetic\_gene\_cluster | 32 | 39.9061032864 | 59 | 8.6e-09 |
